# Analysis of T-cell alloantigen response *via* a direct pathway in kidney transplant recipients with donor-specific antibodies

**DOI:** 10.3389/fimmu.2023.1164794

**Published:** 2023-05-03

**Authors:** Naoya Iwahara, Kiyohiko Hotta, Daiki Iwami, Tatsu Tanabe, Yuka Tanaka, Yoichi M. Ito, Takuya Otsuka, Sachiyo Murai, Yusuke Takada, Haruka Higuchi, Hajime Sasaki, Takayuki Hirose, Hiroshi Harada, Nobuo Shinohara

**Affiliations:** ^1^ Department of Urology, Hokkaido University Hospital, Sapporo, Japan; ^2^ Division of Renal Surgery and Transplantation, Jichi Medical University, Shimotsuke, Tochigi, Japan; ^3^ Data Science Center, Promotion Unit, Institute of Health Science Innovation for Medical Care, Hokkaido University Hospital, Sapporo, Hokkaido, Japan; ^4^ Department of surgical pathology, Hokkaido University Hospital, Sapporo, Hokkaido, Japan; ^5^ Departments of Kidney Transplant Surgery, Sapporo City General Hospital, Sapporo, Hokkaido, Japan

**Keywords:** donor-specific antibody, direct alloantigen recognition pathway, immune monitoring, mixed lymphocyte reaction, kidney transplantation

## Abstract

Donor-specific antibodies (DSAs) are the main cause of graft loss over time. The direct pathway of alloantigen recognition is important in the pathogenesis of acute rejection. Recent studies have suggested that the direct pathway also contributes to the pathogenesis of chronic injury. Nevertheless, there are no reports on T-cell alloantigen response *via* the direct pathway in kidney recipients with DSAs. We analyzed the T-cell alloantigen response *via* the direct pathway in kidney recipients with DSAs (DSA+) or without DSAs (DSA−). A mixed lymphocyte reaction assay was implemented to assess the direct pathway response. DSA+ patients showed significantly higher CD8^+^ and CD4^+^ T cell responses to donor cells than DSA− patients. Furthermore, proliferating CD4^+^ T cells showed a marked increase in Th1 and Th17 responses in DSA+ patients than in DSA− patients. In a comparison between anti-donor and third-party responses, the anti-donor CD8^+^ and CD4^+^ T cell response was significantly lower than the anti-third-party response. In contrast, the donor-specific hyporesponsiveness was absent in DSA+ patients. Our study demonstrated that DSA+ recipients have a greater potential for developing immune responses against the donor tissues *via* the direct alloantigen recognition pathway. These data contribute to an understanding of DSAs pathogenicity during kidney transplantation.

## Introduction

The development of immunosuppressants has reduced acute rejection and improved short-term outcomes after kidney transplantation. Donor-specific antibodies (DSAs) are associated with antibody-mediated rejection (AMR), which leads to poor outcomes. In particular, chronic active antibody-mediated rejection (CAAMR) induced by DSAs is the main cause of graft loss in the long term (1, 2). However, the exact role of DSAs in transplant immunity remains poorly understood.

T cells play a key role in the alloimmune response. Alloreactive T cells recognize HLA-mismatched tissue *via* two different pathways. The direct pathway is important in the pathogenesis of acute rejection in the early post-transplantation period (3). The mixed lymphocyte reaction (MLR) assay, which is a method to measure T-cell alloreactivity *via* the direct pathway has been used as a reliable monitoring system to predict acute rejection (4). In contrast, the indirect pathway of alloantigen recognition plays a pivotal role in late graft failure (5, 6). In addition, the activation of CD4^+^ T cells *via* the indirect pathway is critical for the generation of DSAs (7, 8). Therefore, several studies have focused on the indirect pathway in patients with chronic injury of AMR (9–12). However, recent studies have suggested that the direct pathway also contributes to the pathogenesis of chronic injury (13, 14). Nonetheless, to our knowledge, no study has evaluated the direct pathway of DSAs in patients with kidney transplants.

In this study, our aim was to investigate T-cell alloreactivity *via* the direct pathway in patients with DSAs undergoing kidney transplantation. We analyzed the T-cell alloantigen response *via* the direct pathway in kidney transplant recipients whose graft status was evaluated using graft biopsy.

## Materials and methods

### Patients and samples

In total, 96 patients who underwent a human leukocyte antigens (HLA) incompatible kidney transplantation between 1999 and 2020 were enrolled in the study. The inclusion criteria were functional longstanding living-donor kidney transplants for at least a year after transplantation. Among these recipients, 68 patients without donor-specific antibodies showed stable renal function (DSA− group). Twenty-eight patients were positive for donor-specific antibodies (DSA+ group). Patients were typically maintained on a triple immunosuppressive therapy, including calcineurin inhibitors (tacrolimus or cyclosporine), mycophenolate mofetil, and methylprednisolone. Some patients were subjected to steroid withdrawal and/or everolimus addition based on clinical assessment.

Isolated peripheral blood mononuclear cells (PBMCs) were collected in heparinized tubes from kidney transplant recipients, donors and third parties. Third party cells were obtained from patients in our laboratory who had previously been HLA typed. They were HLA mismatched with each recipient.

The study was approved by the Institutional Review Board of Hokkaido University Hospital (protocol 016-0389) and Sapporo City General Hospital (protocol R1-059-627). Written informed consent was obtained from all patients.

All blood samples from kidney transplant recipients were collected at the time of kidney allograft biopsy.

### Assessment of DSA

Luminex single antigen beads assays (One Lambda, Canoga Park, CA, USA) were performed each year after kidney transplantation to identify donor-specific HLA class I and II antibodies. We defined DSA positivity as a mean fluorescence intensity greater than 1000.

### Biopsy assessment

Protocol biopsies were performed each year after kidney transplantation, if possible. Some patients underwent kidney allograft biopsies owing to the development of *de novo* DSAs. All biopsy specimens were evaluated using light microscopy and C4d immunofluorescence staining.

### Flow cytometry

Isolated PBMCs were analyzed by cell-surface staining using fluorescent antibodies against CD3 (SP34-2 and SK7), CD4 (L200), CD8 (SK1), CD19 (SJ25C1), CD24 (ML5), CD25 (M-A251), CD27 (M-T271), CD28 (CD28.2), CD38 (HIT2), CD45RO (UCHL1), CD56 (B159), CD57 (NK-1), CD69 (FN50), CD138 (MI15), PD1 (NAT105), HLA-DR (G46-6) (BD Pharmingen, San Diego, CA, USA), and CCR7 (G043H7) (BioLegend, San Diego, CA, USA). To assess the intracellular protein expression of FOXP3, INF-γ, IL-4, and IL-17A, the cells were permeabilized using the Foxp3/Transcription Factor Staining Buffer Set (eBioscience, San Diego, CA, USA) and stained with fluorescent antibodies against FOXP3 (PCH101, eBioscience), IFN- γ (B27, BD Pharmingen), IL-4 (8D4-8, BD Pharmingen), and IL-17A (eBio64DEC17, eBioscience). The cells were analyzed on the FACSverse (BD Biosciences, San Diego, CA, USA), and FlowJo software (BD Biosciences) was used for data analysis.

### MLR assay

Responder T cells from recipients were purified by negative selection with an EasySep Human T Cell Isolation Kit (STEMCELL Technologies, Vancouver, BC, Canada). The isolated T cells were labeled with 3 μM carboxyfluorescein diacetate succimidyl ester (CFSE) (Invitrogen, Waltham, MA, USA) at 37°C for 5 min in a 5% CO2 incubator. Labeling of the T cells was terminated by adding cold phosphate-buffered saline containing 2% fetal bovine serum (Biological Industries, Cromwell, CT, USA). Stimulator PBMCs from donor or third parties were irradiated at 30 Gy. Responder T cells were cultured in 96-well U-bottom plates with the stimulator PBMCs at a 1:1 ratio. After 5 days, the cells were harvested and stained with antibodies, and the diluted CFSE signal was analyzed using flow cytometry. For intracellular cytokine staining, Leukocyte Activation Cocktail (BD Pharmingen) was added to the samples per the manufacturer’s protocol, followed by an additional incubation for 4 h.

### Statistical analysis

Statistical analyses were performed using GraphPad Prism 8 (GraphPad Software, Inc.; San Diego, CA, USA). Categorical variables were compared using the chi-square test, and Student’s *t*-test was used for continuous variables. To analyze the number of B cells and B-cell subsets, log transformations were used to reduce skewness. Results with P <0.05 were considered significant.

## Results

### Clinical characteristics of the patients

The patient characteristics of the two groups are summarized in **Table 1**. Age, sex, and the number of ABO-incompatible or HLA mismatches were not significantly different between the groups. All patients received calcineurin inhibitors and mycophenolate mofetil as maintenance immunosuppressants. The majority of DSA+ patients were treated with three or more immunosuppressants. An analysis of immunosuppressant blood levels showed no difference in the area under the curve for the use of tacrolimus and mycophenolate mofetil. The estimated glomerular filtration rate was significantly lower in the DSA+ group than in the DSA− group, which was indicative of graft damage induced by the DSAs or related immune responses. Additionally, total urinary protein/creatinine level was significantly higher in the DSA+ group than in the DSA- group. The time after transplantation was not significantly different between the groups. Among the patients in the DSA+ group, 3 had pre-formed DSAs and 25 developed *de novo* DSAs. None of the patients in the DSA− group showed incidents of rejection, whereas three DSA+ patients developed acute antibody-mediated rejection (AAMR), 17 DSA+ patients developed CAAMR, and 8 DSA+ patients had not yet shown indications of rejection of their kidney grafts.

**Table 1:** Baseline characteristics.

	DSA -	DSA +	P-value
Number	68	28	
Age, years (SD)	52.3 (16.7)	47.8 (16.0)	0.2305
Sex, male / female	36 / 32	15 / 13	0.9551
Primary renal disease			0.9548
Glomerular	25	12	
DMN	11	5	
ADPKD	10	3	
CAKUT	7	2	
Others	15	6	
Number of transplants, 1 / 2	66 / 2	27 / 1	0.8718
ABO incompatible	22	6	0.2845
Mismatches			
HLA - A / B (SD)	2.3 (0.9)	2.2 (1.0)	0.5458
HLA - DR / DQ (SD)	2.4 (1.1)	2.1 (0.9)	0.3574
DSA			
Preformed	0	3	0.0061
De novo	0	25	<0.0001
Pathology			<0.0001
AAMR	0-	3	
CAAMR	0	17	
No rejection	68	8	
Previous history of AR	16	14	0.0110
Immunosuppressant			0.0002
CNI+MMF+MP	49	12	
CNI+MMF+MP+EVR	2	7	
CNI+MMF+EVR	2	5	
CNI+MMF	15	4	
(TAC : CYA)	(57 : 11)	(28 : 0)	0.0237
AUC			
TAC AUC_0-24_ (ng•hr / ml) (SD)	125.4 (30.8)	112.6 (35.5)	0.0929
CyA AUC_0-12_ (ng•hr / ml) (SD)	1615.0 (762.2)	–	
MMF AUC_0-12_ (μg•hr / ml) (SD)	49.8 (15.3)	53.8 (18.5)	0.1660
eGFR (ml / min /1.73m^2^) (SD)	52.4 (12.1)	41.4 (18.3)	0.0008
UP/ Ucr (g / gCr) (SD)	0.2 (0.3)	1.0 (1.6)	0.0001
Time after transplants, years (SD)	6.6 (5.1)	8.7 (5.9)	0.0789

DSA, donor-specific antibody; DMN, diabetic nephropathy; ADPKD, autosomal dominant polycystic kidney disease; CAKUT, congenital anomalies of the kidney and urinary tract; AAMR, acute antibody-mediated rejection; CAAMR, chronic active antibody-mediated rejection; AR, acute rejection: CNI, calcineurin inhibitor; MMF, mycophenolate mofetil; MP, methylprednisolone; EVR, everolimus; TAC, tacrolimus; CyA, cyclosporine A; AUC, area under curve; eGFR, estimated glomerular filtration rate; UP, urinary protein; Cr, creatinine.

Immunological characteristics of DSA+ patients are summarized in **Table 2**. One patient developed both anti-HLA class I and II DSAs, two had anti-HLA class I DSAs, and the remaining patients had anti-HLA class II DSAs. The sum of the mean fluorescence intensity of DSA was 10260 ± 2374 (mean ± SEM). Biopsies from AAMR and CAAMR patients showed microvascular inflammation (glomerulitis and/or peritubular capillaritis score ≥2), while only CAAMR patients showed transplant glomerulopathy (chronic glomerulopathy score >0). In addition, one of three AAMR patients, seven of 17 CAAMR patients, and two of eight patients without rejection had C4d positive in in peritubular capillaries based on immunofluorescence.

**Table 2:** Immunological characteristics of patients with donor-specific antibodies.

Patient	Primary renal disease	Maintenance immunosuppression	DSA class 1(MFI)	DSA class 2 (MFI)	Pathology	Time after transplants (years)
DSA+ 1	Others	CNI+MMF+MP	negative	DRB1 (1231)	AAMR (ptc2, c4d0, g1, cg0)	1
DSA+ 2	DMN	CNI+MMF+MP	negative	DQB1 (18768)	AAMR (ptc1, c4d0, g1, cg0)	2
DSA+ 3	ADPKD	CNI+MMF	negative	DRB4 (4650)	AAMR (ptc2, c4d1, g3, cg0)	4
DSA+ 4	DMN	CNI+MMF+MP+EVR	A2 (1780)	negative	CAAMR (ptc1, c4d0, g2, cg1)	3
DSA+ 5	Glomerular	CNI+MMF	negative	DR1 (1090) DQ5 (11130)	CAAMR (ptc2, c4d0, g3, cg3)	5
DSA+ 6	Others	CNI+MMF+MP+EVR	negative	DQB1 (2420)	CAAMR (ptc1, c4d3, g1, cg1)	5
DSA+ 7	ADPKD	CNI+MMF	negative	DQ6 (2060)	CAAMR (ptc1, c4d0, g2, cg2)	5
DSA+ 8	Others	CNI+MMF+MP+EVR	negative	DQB1 (29823)	CAAMR (ptc1, c4d0, g2, cg2)	6
DSA+ 9	Glomerular	CNI+MMF+EVR	negative	DQ7 (1311)	CAAMR (ptc0, c4d3, g2, cg2)	7
DSA+ 10	DMN	CNI+MMF+MP	negative	DQB1 (4809)	CAAMR (ptc0, c4d3 g0, cg1)	9
DSA+ 11	Glomerular	CNI+MMF+MP	negative	DRB1 (1100)	CAAMR (ptc1, c4d0, g3, cg3)	9
DSA+ 12	Glomerular	CNI+MMF+MP+EVR	negative	DR53 (22295)	CAAMR (ptc2, c4d0, g2, cg1)	13
DSA+ 13	Others	CNI+MMF+MP	negative	DQ7 (3579)	CAAMR (ptc0, c4d3, g1, cg1)	15
DSA+ 14	Glomerular	CNI+MMF+MP	negative	DR51 (9796)	CAAMR (ptc2, c4d3, g1, cg2)	15
DSA+ 15	ADPKD	CNI+MMF+MP	A2 (9006)	DR53 (25450) DQ9 (25133)	CAAMR (ptc1, c4d2, g1.cg3)	15
DSA+ 16	Others	CNI+MMF+EVR	negative	DR53 (10057)	CAAMR (ptc0, c4d0, g3, cg1)	16
DSA+ 17	Glomerular	CNI+MMF+MP	negative	DQA1 (2396)	CAAMR (ptc1, c4d0, g2, cg3)	17
DSA+ 18	Glomerular	CNI+MMF	negative	DR53(4161) DQ3 (17122)	CAAMR (ptc0, c4d0, g2, cg3)	17
DSA+ 19	Others	CNI+MMF+MP	negative	DQ7 (14473)	CAAMR (ptc2, c4d3, g2, cg3)	17
DSA+ 20	CAKUT	CNI+MMF+MP+EVR	negative	DQB1 (21783)	CAAMR (ptc2, c4d0, g2, cg3)	19
DSA+ 21	Glomerular	CNI+MMF+MP	A2 (6765)	negative	No rejection (ptc0, c4d0, g0, cg0)	2
DSA+ 22	Glomerular	CNI+MMF+EVR	negative	DR9 (1136)	No rejection (ptc0, c4d0, g0, cg0)	2
DSA+ 23	Glomerular	CNI+MMF+MP	negative	DQB1 (4987)	No rejection (ptc0, c4d3, g1, cg0)	2
DSA+ 24	DMN	CNI+MMF+EVR	negative	DQ7 (1311)	No rejection (ptc0, c4d0, g0, cg0)	3
DSA+ 25	Glomerular	CNI+MMF+EVR	negative	DR4 (1583)	No rejection (ptc0, c4d3, g0, cg0)	5
DSA+ 26	CAKUT	CNI+MMF+MP	negative	DQB1 (6414)	No rejection (ptc0, c4d0, g1, cg0)	9
DSA+ 27	DMN	CNI+MMF+MP+EVR	negative	DQ4 (14658)	No rejection (ptc0, c4d0, g0, cg1)	10
DSA+ 28	Glomerular	CNI+MMF+MP+EVR	negative	DQ7 (5016)	No rejection (ptc0, c4d0, g0, cg0)	15

DSA, donor-specific antibody; MFI, mean fluorescence intensity; DMN, diabetes nephropathy; ADPKD, autosomal dominant polycystic kidney disease; CAKUT, congenital anomalies of the kidney and urinary tract; CNI, calcineurin inhibitor; MMF, mycophenolate mofetil; MP, methylprednisolone; EVR, everolimus; AAMR, acute antibody-mediated rejection; CAAMR, chronic active antibody-mediated rejection; ptc, peritubular capillaritis score; g, glomerulitis score; cg, chronic glomerulopathy score.

### Immunophenotypes of peripheral lymphocytes

**Figure 1:**
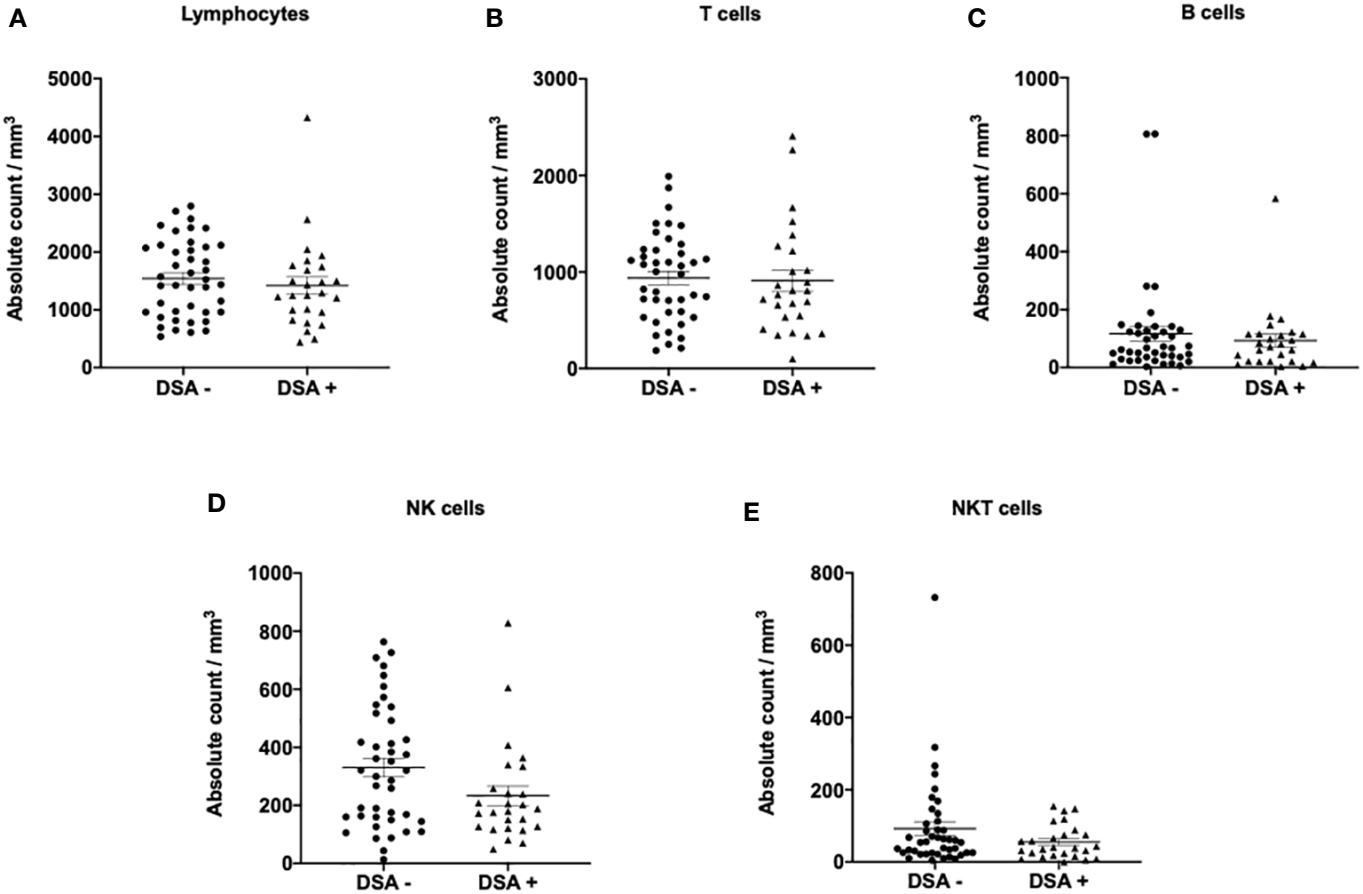
Lymphocyte subsets in patients without donor-specific antibodies (DSA -) and with DSA (DSA+). The number of **(A)** lymphocytes, **(B)** T cells (CD3^+^), **(C)** B cells (CD19^+^), **(D)** NK cells (CD3^-^CD56^+^), and **(E)** NKT cells (CD3^+^CD56^+^). No significant differences in the number of lymphocytes, T, B, NK, and NKT cells were observed in the peripheral blood between DSA- and DSA+ patients. Data are shown as mean ± SEM. An unpaired *t*-test was performed.

**Figure 2:**
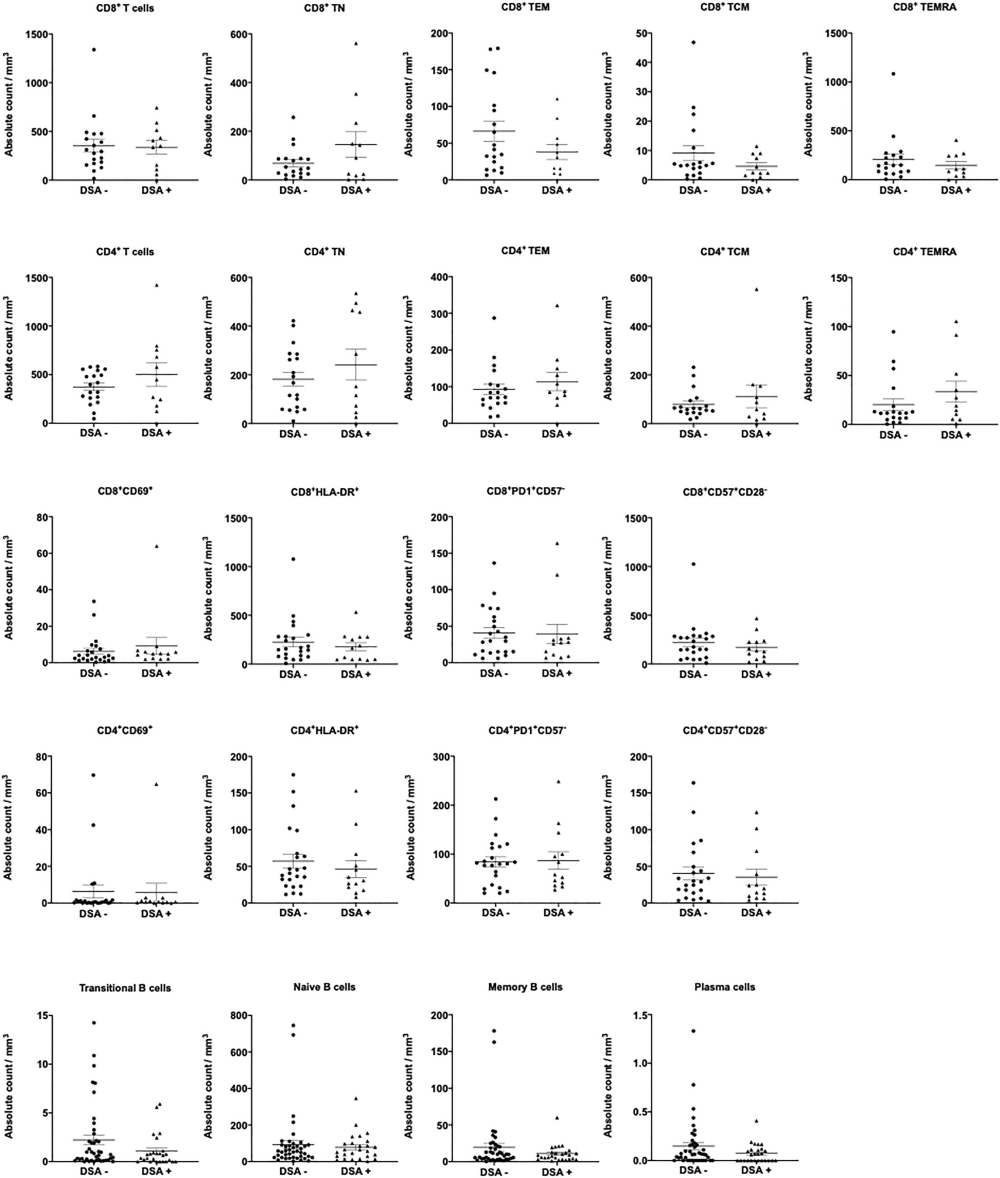
T and B cell subsets in patients without donor-specific antibodies (DSA-) and with DSA (DSA+). The number of T and B cell subsets was analyzed in the peripheral blood between DSA - and DSA + patients. There were no significant differences in T and B cell subsets between DSA- and DSA+ patients. Data are shown as the mean ± SEM. An unpaired *t*-test was performed.

The composition of peripheral blood lymphocytes was characterized in DSA− and DSA+ patients. CD3, CD19, and CD56 expression levels were monitored using flow cytometry, and no significant differences in the number of T (CD3^+^), B (CD19^+^), NK (CD3^-^CD56^+^), and NKT (CD3^+^CD56^+^) cells in the peripheral blood between the groups was observed (**Figure 1**). Next, the T and B cell subsets were further analyzed. The CD8^+^ or CD4^+^ T cell subsets were divided into naïve (CCR7^+^CD45RO^−^, TN), central memory (CCR7^+^CD45RO^+^, TCM), effector memory (CCR7^−^CD45RO^+^, TEM), and highly differentiated effector T cells without CD45RO expression that re-expressed CD45RA (CCR7^−^CD45RO^−^, TEMRA) (**Supplemental Figure 1**). We also assessed several more subsets of CD8+ or CD4+ T cells, such as activation (CD69+, and HLA-DR+), exhaustion (PD1+CD57-), and senescence (CD57+CD28-) (**Supplemental Figure 1**). B cell subsets were determined as follows: transitional B cells (CD19^+^CD24^hi^CD38^hi^), naïve B cells (CD19^+^CD24^lo^CD38^lo^), memory B cells (CD19^+^CD27^+^), and plasma cells (CD38^+^CD138^+^) (**Supplemental Figure 1**). However, there were no significant differences in the number of T and B cell subsets between the groups (**Figure 2**).

### Anti-donor CD8^+^ and CD4^+^ T-cell proliferation *via* the direct pathway in DSA+ patients

In a preliminary experiment, we evaluated the T-cell response against donor antigens *via* the direct pathway in 15 DSA− and 5 DSA+ patients. As previously reported in patients with acute rejection, PBMCs from the transplant recipients were labeled with CFSE and cultured with irradiated donor PBMCs for 5 days, after which the cultured cells were stained for CD4 and CD8 and analyzed using flow cytometry. No differences were observed in the anti-donor CD8^+^ and CD4^+^ T cell responses between the groups (**Supplemental Figure 2**). Unlike that in patients with acute cellular rejection, no marked T cell proliferation was observed in DSA+ patients.

**Figure 3:**
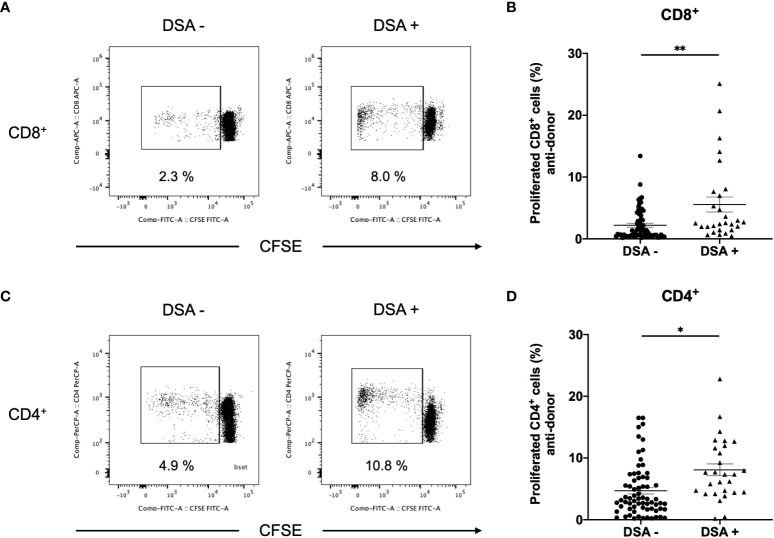
Aq1Anti-donor CD8^+^ and CD4^+^ T cell response in patients without donor-specific antibodies (DSA-) and with DSA (DSA+). The CD3^+^ cells isolated from DSA- and DSA+ patients were labeled with CFSE and were cultured with irradiated donor peripheral blood mononuclear cells for 5 days. The cultured cells were stained for CD4 and CD8. Representative flow cytometric data (**A**: CD8, **C**: CD4) and cell proliferation rate (**B**: CD8, **D**: CD4) are shown. Anti-CD8^+^ and CD4^+^ T-cell hyper-responses were observed in DSA+ patients, and they were significantly higher than those observed in DSA- patients, Data are presented as mean ± SEM. An unpaired *t*-test was performed. *p<0.01, **p<0.001.

To improve the accuracy of T-cell alloreactivity *via* the direct pathway, we used isolated T cells as responders, and this excluded the T cell response *via* the indirect pathway. Anti-donor CD8+ T-cell responses were found to be significantly higher in DSA+ patients than in DSA− patients (**Figures 3A, B**). Similarly, anti-donor CD4+ T-cell responses were significantly higher in DSA+ patients than in DSA− patients (**Figures 3C, D**).

### Anti-donor Th1 and Th17 proliferation *via* the direct pathway in DSA+ patients

**Figure 4:**
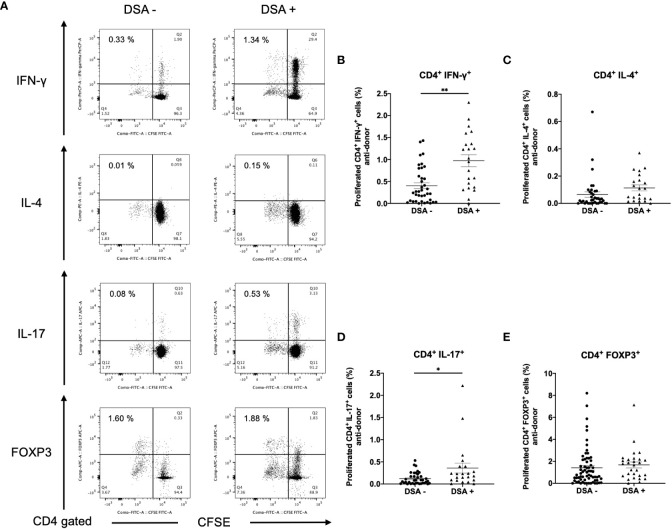
Anti-donor CD4^+^ T cell subset response in patients without donor-specific antibodies negative (DSA-) and with DSA (DSA+). T cells isolated from DSA- and DSA+ patients were labeled with CFSE and were cultured with irradiated donor peripheral blood mononuclear cells for 5 days. The cultured cells were stained for CD4, IFN-γ, IL-4, IL-17 and FOXP3. Representative flow cytometric data **(A)** and cell proliferation rate (**B**: CD4^+^IFN- γ ^+^, C: CD4^+^IL-4^+^, D: CD4^+^IL-17^+^, E: CD4^+^FOXP3^+^) are shown. Proliferating CD4^+^ cells showed a marked increase in Th1 (CD4^+^IFN- γ^+^) and Th17 (CD4^+^IL-17^+^) response in DSA+ compared with DSA- those in patients **(B, D)**. However, there were no differences in donor-reactive Th2 (CD4^+^IL-4^+^) or Treg (CD4^+^FOXP3^+^) cells between the groups **(C, E)**. Data are presented as the mean ± SEM. An unpaired *t*-test was performed. *p<0.01, **p<0.0001.

To identify CD4^+^ T cell subsets that showed expansion, responder T cells from DSA− and DSA+ patients were stained for INF- γ, IL-4, IL-17, and FOXP3 (**Figure 4A**). Although no differences were observed in the peripheral blood number of Th1 (CD4^+^INF-γ^+^) and Th17 (CD4^+^IL-17^+^) between DSA- and DSA+ patients (**Supplemental Figure 3**), proliferating CD4^+^ T cells showed a marked increase in the Th1 (CD4^+^INF- γ^+^) and Th17 (CD4^+^IL-17^+^) response in DSA+ patients compared with that in the DSA− patients (**Figures 4B, D**). In contrast, there were no differences in donor reactive Th2 (CD4^+^IL4^+^) or Treg (CD4^+^FOXP3^+^) cells between the groups (**Figures 4C, E**). These results suggest that DSAs are associated with the response of the classical proinflammatory Th1 and Th17 cells.

**Figure 5:**
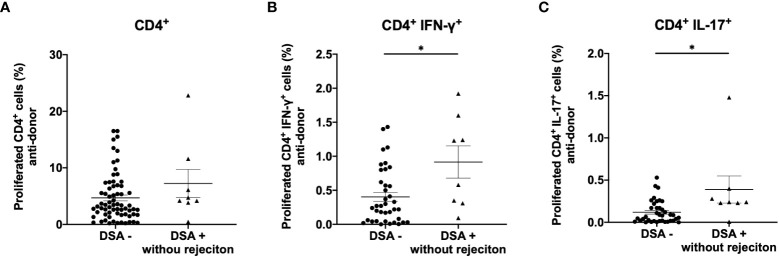
Analysis of anti-donor CD4^+^ T-cell response in patients with donor-specific antibodies without rejection (DSA+ without rejection). Anti-donor CD4^+^ T cell response was comparable between DSA+ without rejection and DSA- patients **(A)**. However, a significantly higher proportion of the proliferating CD4^+^ T cells showed a Th1 (CD4^+^IFN- γ^+^) and Th17 (CD4^+^IL-17^+^) phenotype in the DSA+ patients without rejection than in DSA- patients **(B, C)**. Data are shown as the mean ± SEM. An unpaired *t*-test was performed. *p<0.01.

We also explored the relationship between DSAs and the direct pathway response in rejection-free patients. Eight of the 28 patients with DSAs showed no transplant rejection (DSA+ without rejection). The anti-donor CD4^+^ T cell response was comparable between DSA+ patients without rejection and DSA− patients (**Figure 5A**). However, Th1 and Th17 responses were significantly higher in DSA+ patients without rejection than in DSA− patients (**Figures 5B, C**).

### Comparison between the anti-donor and third-party responses

**Figure 6:**
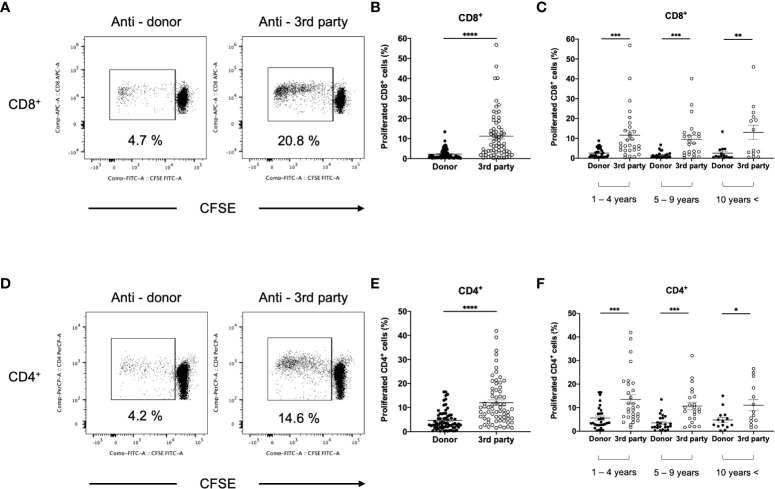
Comparison between anti-donor and third-party response in patients without donor-specific antibodies (DSA-). The isolated T cells from DSA- patients were labeled with CFSE and were cultured with irradiated donor or third-party peripheral blood mononuclear cells for 5 days. The cultured cells were stained for CD8 and CD4. Representative flow cytometric data (**A**: CD8, **D**: CD4) and proliferated cells rate (**B**: CD8, **E**: CD4) are shown. The response at 1-4, 5-9, and more than 10 (10 years <) years after transplantation is shown (**C**: CD8, **F**: CD4). Anti-donor CD8^+^ and CD4^+^ T-cell responses were significantly lower than the anti-third-party response **(B, E)**. This donor-specific T-cell hyporesponsiveness persisted for 10 years after transplantation **(C, F)**. Data are presented as the mean ± SEM. A paired *t*-test was performed. *p<0.05, **p<0.01, ***p<0.001, ****p<0.0001.

To compare anti-donor and third-party responses, T cells isolated from DSA− and DSA+ patients were stimulated with third-party PBMCs. We hypothesized that immunosuppressant treatments can decrease the lymphocyte response to both donor and the third-party in kidney transplant patients. Interestingly, the anti-donor CD8^+^ and CD4^+^ T cell responses were significantly lower than the anti-third-party response in DSA− patients (**Figures 6A, B, D, E**). These results indicate that donor-specific hyporesponsiveness is observed in CD8^+^ and CD4^+^ T cells from DSA− patients and persists for 10 years after transplantation (**Figures 6C, F**).

**Figure 7:**
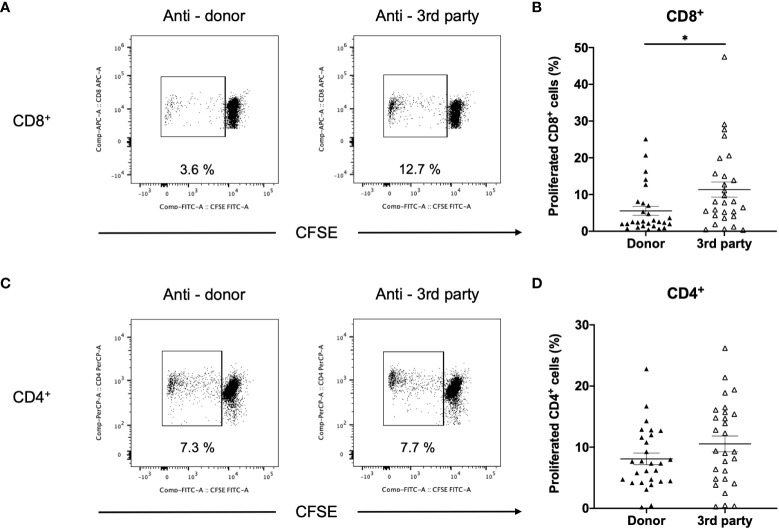
Comparison between anti-donor and third-party responses in patients with donor-specific antibodies (DSA+). T cells isolated from (DSA+) patients were labeled with CFSE and were cultured with irradiated donor or third-party peripheral blood mononuclear cells for 5 days. The cultured cells were stained for CD8 and CD4. Representative flow cytometric data (**A**: CD8, **C**: CD4) and proliferated cell rate (**B**: CD8, **D**: CD4) are shown. Anti-donor CD8^+^ T-cell response was significantly lower than the third-party response **(B)**. However, CD4^+^ T-cell proliferation was comparable in terms of donor and third-party responses **(D)**. Data are presented as mean ± SEM. A paired *t-*test was performed. *p<0.05.

In DSA+ patients, the anti-donor CD8+ T-cell response was significantly lower than the third-party response (**Figures 7A, B**). In contrast, CD4^+^ T cell proliferation was similar in the donor and third-party (**Figures 7C, D**), indicating that donor-specific hyporesponsiveness of CD4^+^ T cells was absent in DSA+ patients.

## Discussion

DSAs are a major risk factor of CAAMR and graft loss (1, 2). The importance of DSAs in kidney transplantation has been recognized for over 50 years (15). However, little is known about how DSAs affect the kidney allograft *via* direct pathway. Therefore, we explored the direct-pathway T-cell responses in 96 kidney transplant patients. Patients with DSAs displayed higher CD8^+^ and CD4^+^ responses to donors than those without DSAs. Moreover, activated CD4^+^ T cells showed a marked increase in Th1 and Th17 responses in patients with DSAs. Furthermore, no rejection patients with DSAs showed increased anti-donor Th1 and Th17 responses. We further demonstrated that donor-specific hyporesponsiveness of CD8^+^ and CD4^+^ T cells was observed in patients without DSAs, but the hyporesponsiveness of CD4^+^ T cells was absent in patients with DSAs.

T-cell allorecognition is still considered critical for short- and long-term outcomes of kidney transplantation. Alloreactive T cells recognize HLA-mismatched tissue *via* direct and indirect pathways. In the direct pathway, T cells recognize non-self MHC molecules present on the surface of donor cells. The direct pathway was regarded as short-lived and responsible for acute rejection in the early transplant period (3). The MLR assay is a method for measuring T cell alloreactivity *via* the direct pathway and has been widely used as a reliable monitoring system for acute T cell-mediated rejection in clinical transplantation (4, 16–18). We first performed the CFSE/MLR assay using PBMCs as responders and found that this assay could not distinguish between DSA+ and DSA− patients. Therefore, we used isolated T cells as responders, which exclude the T cell response *via* the indirect pathway (19). We identified that anti-donor CD8^+^ and CD4^+^ T-cell responses in DSA+ patients were significantly higher than those in DSA− patients. Therefore, the use of isolated T cells from transplant patients as responders revealed the activation of T cells *via* the direct pathway of DSAs. It is noteworthy that our study is the first to evaluate T-cell alloreactivity *via* the direct pathway of DSAs in clinical kidney transplant recipients.

Next, we focused on the differences in the CD4^+^ T cell phenotype between DSA+ and DSA− patients. Our study revealed a marked increase in Th1 and Th17 responses in DSA+ patients. The data suggest that Th1 and Th17 responses were activated in kidney transplant recipients with DSAs. Several studies have reported an association between CAAMR and Th1 and Th17 responses. Homs et al. reported that INF- γ and the transcription factor T-bet (both functionally defined markers of Th1 CD4 T cells) are more strongly expressed in the allografts of patients with CAAMR than in normal control patients and those without CAAMR (20). Several studies also showed an increased prevalence of the Th17-cell phenotype in kidney transplant recipients with CAAMR. Furthermore, Th17 immunity contributes to chronic allograft rejection in patients with lung and heart transplants (21–23). In our study, we performed a comparative analysis of different subsets of CD4^+^ T cells by staining responder T cells for INF-γ, IL-4, IL-17, and FOXP3. We observed a marked increase in Th1 and Th17 responses in DSA+ patients. A previous study reported that the decreased frequency of regulatory T cells in peripheral blood was associated with CAAMR in kidney recipients (22, 23); however, no differences in Treg expansion between DSA− and DSA+ recipients were observed.

We also found significantly higher Th1 and Th17 expansion *via* the direct pathway in rejection-free patients with DSAs. A recent study showed that transitional B cell frequencies were higher in rejection-free patients with *de novo* DSAs than in those without *de novo* DSAs (24). DSAs are associated with CAAMR, which is the main cause of graft loss in the long term. DSAs are considered a trigger for CAAMR. Therefore, rejection-free patients with DSAs might eventually develop CAAMR. This result indicates that monitoring Th1 and Th17 responses could identify patients with the potential for developing CAAMR. The identification of transplant patients with the potential for CAMMR permits therapeutic interventions that can be administered before histological changes occur in the graft tissue. Early interventions (plasmapheresis, pulse steroid, intravenous immunoglobulin, and rituximab) improve clinical outcomes in subclinical antibody-mediated rejection (25–27). Thus, prediction of the CAAMR incidence might prevent or delay CAAMR development. However, the number of DSA+ patients without rejection was small, and it has not been confirmed that they will develop CAAMR in the future. We must conduct long-term studies for confirmation.

Donor-specific hyporesponsiveness in CD4^+^ and CD8^+^ T cell responses was observed in DSA− patients during immunosuppressant therapy. A similar response has been reported in kidney recipients with induced immune tolerance from combined kidney and bone marrow transplantation (28). It has been suggested that the mechanism of donor-specific hyporesponsiveness and tolerance induces the clonal deletion of donor-reactive T cells (29). However, this phenomenon is controversial in solid organ recipients who receive immunosuppressive therapy. A recent study showed that donor-specific hyporesponsiveness occurs in both liver-kidney and solitary liver transplant patients, but this response was not observed in kidney recipients (30). Additionally, a few studies reported this hyporesponsiveness in kidney recipients with a stable renal function; however, recent studies were inconclusive because of the small number of patients and short follow-up periods (16, 31). This study is the first to clarify this phenomenon in kidney graft recipients using an adequate number of patients. In addition, we discovered that the donor-specific hyporesponsive state persist in the long term after transplantation. Meanwhile, we found that donor-specific hyporesponsiveness of CD4^+^ T cells was absent in DSA+ patients. Poggio et al. reported that ratios of donor/third-party enzyme-linked immunosorbent spot responses increased in chronic allograft nephropathy (CAN) (32). Additionally, CAN resulted in the development of DSAs to a greater degree than that in the control group. As the production of DSAs requires interactions between B cells and CD4^+^ T cells, we could detect the activation of donor-specific CD4^+^ T cell responses.

The strength of our study is that we analyzed a large number of patients during a long-term follow-up period. Furthermore, the overall status of the transplanted kidney was accurately evaluated in all recipients using graft biopsy. Nonetheless, there are some limitations to this study. First, we conducted a cross-sectional study and did not serially analyze the immune status of the recipients. Therefore, longitudinal and prospective studies that include monitoring patients before transplantation are ongoing. Second, our study did not include an independent validation cohort. Third, donor blood samples are required for our MLR assay. Thus, when we serially perform the MLR assay, we must collect blood samples from the donor several times. Currently, we are developing a new technique to increase the number of donor cells through incubation. If this technique is established successfully, then the donor blood will only need to be collected once for use at different time points. Fourth, we evaluated the direct allo-response *in vitro* assay using antigen-presenting cells (APCs) from donors in this study. However, the *in vivo* setting, viable APCs of donor origin are lacking after 1 year of transplantation. Thus, the more accurate *in vitro* correlate of the long-term transplant patient’s status is considered not the direct pathway but rather a pathway through recipient APCs cross-dressed with donor MHC molecules, termed the semi-direct pathway. Recent rodent studies indicated that the presentation of intact donor MHC molecules by donor APCs would lead to results similar to those with presentation of the same intact allogeneic MHC molecules by recipient APCs. Therefore, our MLR assay is certainly a rational and useful methods to assess T-cell alloresponse in clinical settings (33, 34).

In conclusion, our study demonstrated that DSA+ recipients have a greater potential of developing immune responses against the donor tissues *via* the direct alloantigen recognition pathway. These data contribute to an understanding of DSA pathogenicity during kidney transplantation.

## Data availability statement

The original contributions presented in the study are included in the article/**Supplementary Material**. Further inquiries can be directed to the corresponding author.

## Ethics statement

The studies involving human participants were reviewed and approved by the Institutional Review Board of Hokkaido University Hospital (protocol 016-0389) and Sapporo City General Hospital (protocol R1-059-627). The patients/participants provided their written informed consent to participate in this study.

## Author contributions

NI performed the experiments, analyzed the data, and wrote the manuscript. KH designed and supervised the experiments, analyzed the data, and wrote the manuscript. YTan performed the experiments. TT, SM, YTak, HHi, HS, TH, and HHa acquired the data. YI contributed to statistical design. TO performed the pathological analysis. DI and NS supervised the experiments. All authors contributed to the article and approved the submitted version.
