# Supplementary figures and images for: Analysis of T-cell alloantigen response via a direct pathway in kidney transplant recipients with donor-specific antibodies
Source: Front Immunol. 2023 May 3;14:1164794. doi: 10.3389/fimmu.2023.1164794 (PMC10189043; doi:10.3389/fimmu.2023.1164794)

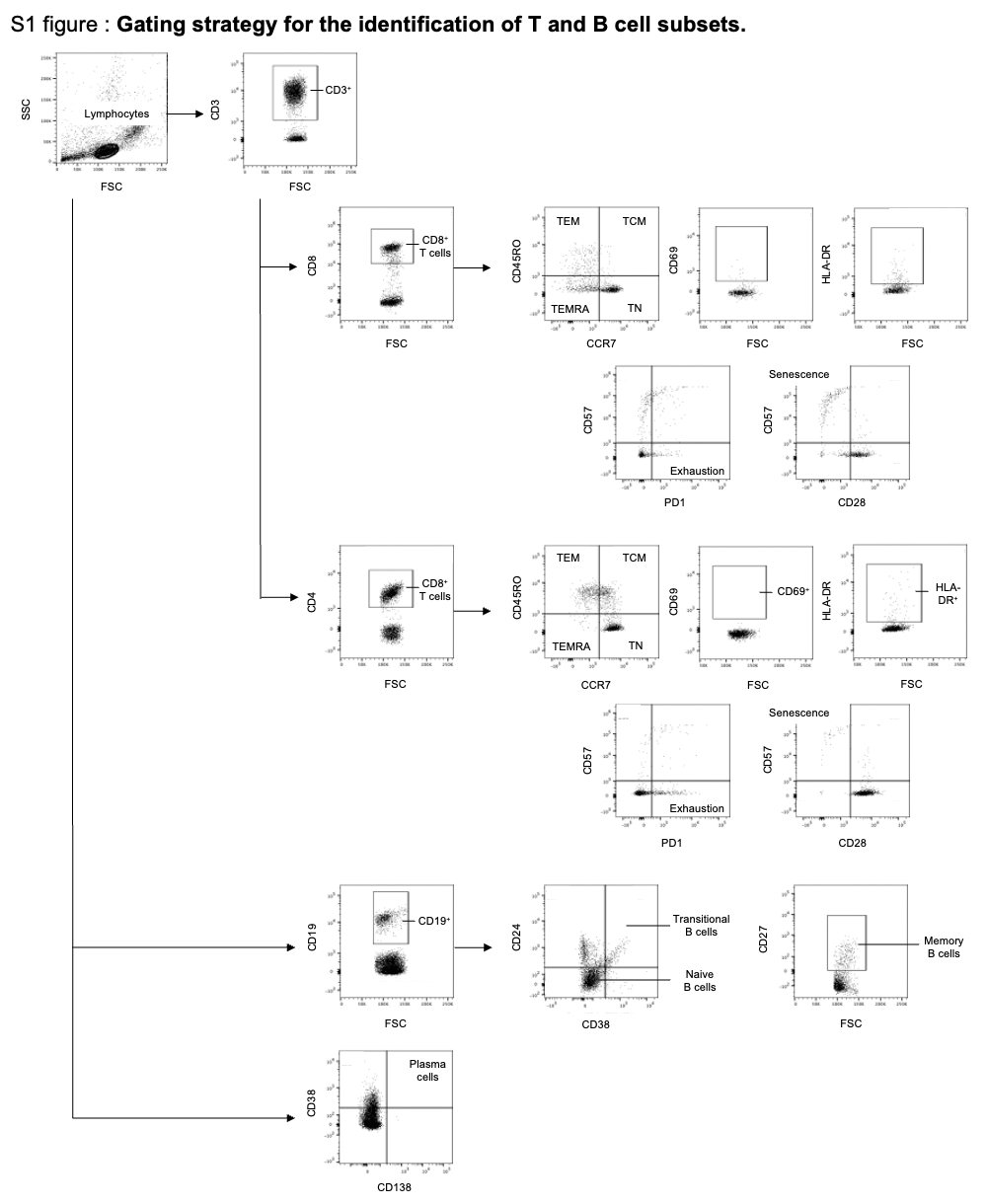

Supplement: Supplementary file 1 [file Image_1.tiff]

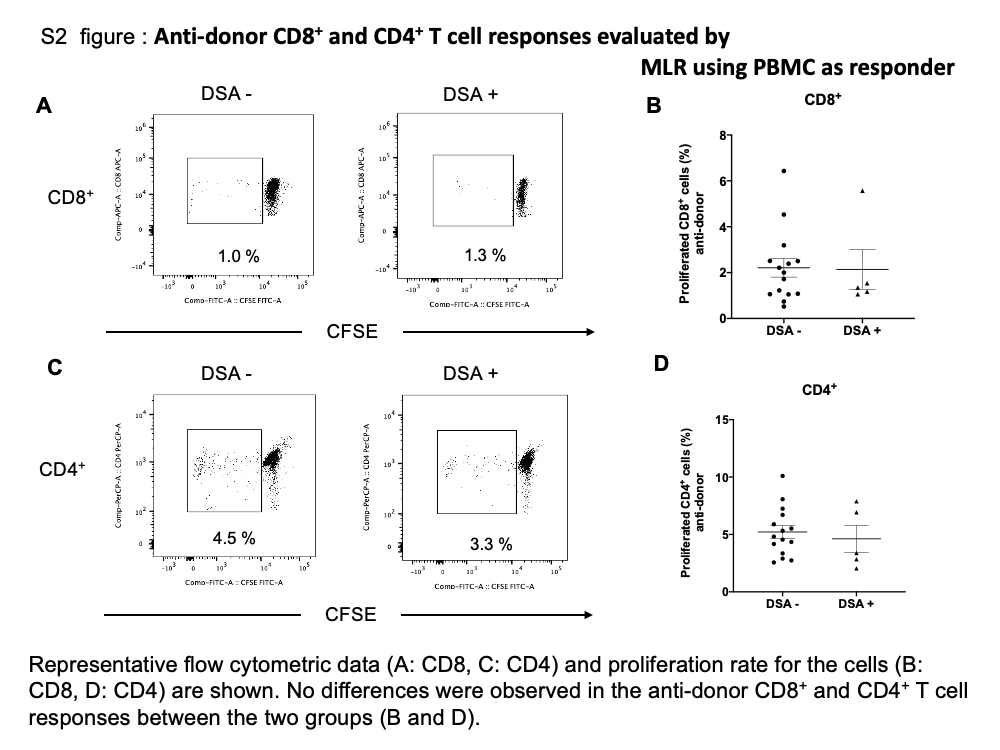

Supplement: Supplementary file 2 [file Image_2.tiff]

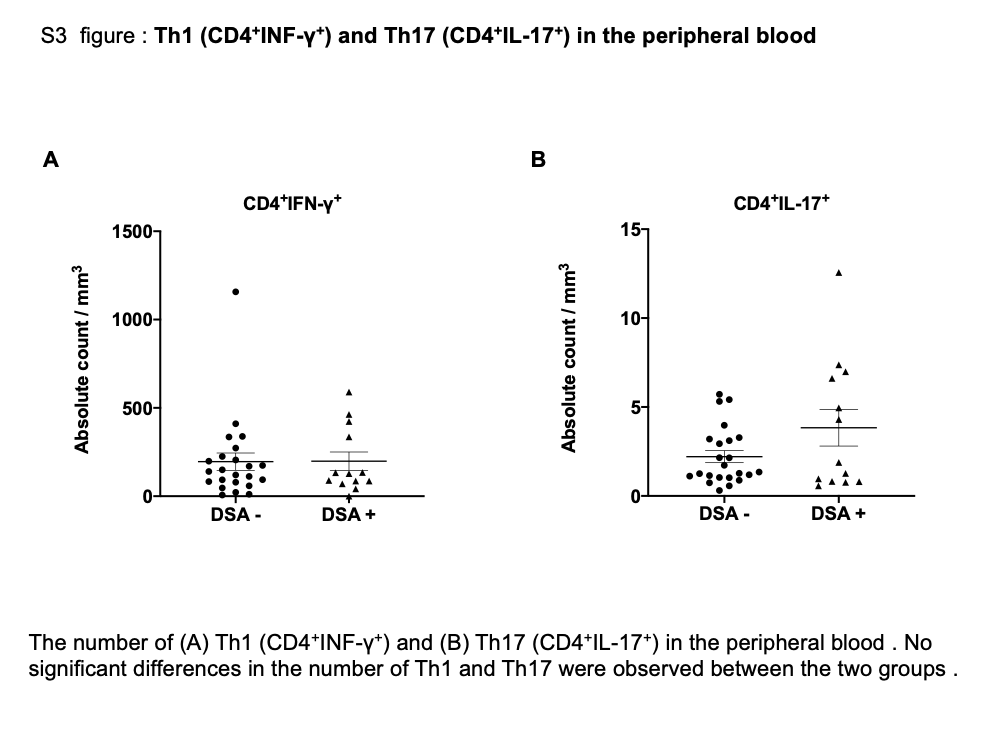

Supplement: Supplementary file 3 [file Image_3.tiff]
